# Controllable Deposition of Bi onto Pd for Selective Hydrogenation of Acetylene

**DOI:** 10.3390/molecules28052335

**Published:** 2023-03-03

**Authors:** Hongquan Kang, Jianzhou Wu, Baohui Lou, Yue Wang, Yilin Zhao, Juanjuan Liu, Shihui Zou, Jie Fan

**Affiliations:** 1 Key Laboratory of Applied Chemistry of Zhejiang Province, Department of Chemistry, Zhejiang University, Hangzhou 310027, China; 2 College of Materials & Environmental Engineering, Hangzhou Dianzi University, Hangzhou 310036, China; 3 Shanxi-Zheda Institute of Advanced Materials and Chemical Engineering, Taiyuan 030032, China

**Keywords:** acetylene hydrogenation, active sites, selective deposition, palladium, bismuth

## Abstract

The rational regulation of catalyst active sites at atomic scale is a key approach to unveil the relationship between structure and catalytic performance. Herein, we reported a strategy for the controllable deposition of Bi on Pd nanocubes (Pd NCs) in the priority order from corners to edges and then to facets (Pd NCs@Bi). The spherical aberration-corrected scanning transmission electron microscopy (ac-STEM) results indicated that Bi_2_O_3_ with an amorphous structure covers the specific sites of Pd NCs. When only the corners and edges of the Pd NCs were covered, the supported Pd NCs@Bi catalyst exhibited an optimal trade-off between high conversion and selectivity in the hydrogenation of acetylene to ethylene under ethylene-rich conditions (99.7% C_2_H_2_ conversion and 94.3% C_2_H_4_ selectivity at 170 °C) with remarkable long-term stability. According to the H_2_-TPR and C_2_H_4_-TPD measurements, the moderate hydrogen dissociation and the weak ethylene adsorption are responsible for this excellent catalytic performance. Following these results, the selectively Bi-deposited Pd nanoparticle catalysts showed incredible acetylene hydrogenation performance, which provides a feasible perspective to design and develop highly selective hydrogenation catalysts for industrial applications.

## 1. Introduction

The selective hydrogenation of acetylene to ethylene is essential for the industrial removal of trace acetylene impurities in an ethylene-rich stream produced from the cracking process [1,2,3,4]. Herein, two key issues should be addressed. One is that the included acetylene can deactivate the Ziegler–Natta catalysts during subsequent polymerization [5]. The other is that the over–hydrogenation of ethylene to ethane greatly reduces the commercial value. Owing to its high hydrogenation activity, Pd is regarded as the most optimum candidate for acetylene hydrogenation [6]. However, Pd-based catalysts usually suffer from low selectivity to ethylene, especially at high acetylene conversions, and poor long–term stability due to the formation of green oil. The poor selectivity of Pd catalysts is due to the superior hydrogen dissociation capacity and strong ethylene adsorption [7].

Numerous efforts, including site-isolation strategies and local environmental regulations for regulating the electronic and/or geometric structures of Pd, have been made to improve the selectivity by incorporating a second metal to form Pd-based intermetallic compounds, alloys and single–atom catalysts [8,9,10,11,12,13,14,15,16,17,18,19]. However, these strategies are mostly achieved at the expense of catalytic activity. More importantly, due to the limitations of synthetic methods, the regulation of Pd active sites is usually accomplished through non-selective doping, which results in an excessive poisoning of the active sites. Consequently, it remains challenging to selectively regulate the Pd active sites for an optimum catalytic performance because of the inherent trade-off between high catalytic activity and selectivity.

Acetylene semi-hydrogenation over Pd catalysts is well-known to be structure-sensitive [20]. Different types of active sites, including corners, edges and facets, have diverse effects on the catalytic performance [21,22,23,24]. To guide the design of catalysts with a high catalytic performance, it is crucial to precisely regulate the active sites and selectively expose specific active sites to gain an in-depth understanding toward the structure-activity relationship of the Pd catalysts.

Bismuth-modification has recently been reported as an effective strategy to improve the selective hydrogenation performance of Pd catalysts [25,26,27,28]. In this work, we have achieved the selective deposition of Bi on Pd nanocubes (Pd NCs) from the corners to the edges and then to the facets (Pd NCs@Bi) by regulating the Bi-to-Pd molar ratio. The as-synthesized nanocrystals were further loaded on *α*-Al_2_O_3_ to form the Pd NCs@Bi/Al_2_O_3_ catalysts. The selective deposition on the corners and edges of Pd NC endows the catalyst with the unique properties of moderate hydrogen dissociation and weak ethylene adsorption, leading to an optimal trade-off between high conversion and selectivity for selective acetylene hydrogenation. The catalyst exhibits 94.3% C_2_H_4_ selectivity at 99.7% C_2_H_2_ conversion and good long-term stability under the simulated reaction conditions of front-end acetylene hydrogenation.

## 2. Results and Discussion

### 2.1. Synthesis and Structural Characterizations

**Figure 1:**
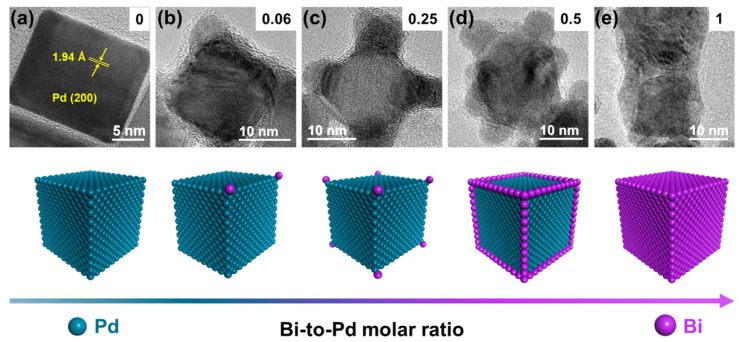
Selective deposition of Bi on Pd nanocubes (Pd NCs@Bi). HRTEM images of (**a**) Pd NCs, (**b**) Pd NCs@Bi_0.06_, (**c**) Pd NCs@Bi_0.25_, (**d**) Pd NCs@Bi_0.5_, (**e**) Pd NCs@Bi_1_. The illustrations in the upper right corners represent the Bi-to-Pd molar ratio. The diagram below represents corresponding models of Pd NCs@Bi.

Pd NCs@Bi*_x_* (*x* is the Bi-to-Pd molar ratio) were synthesized by a seed growth method, illustrated in Figure S1. Schematically, Pd NCs with an average size of 20 nm were synthesized via the liquid phase synthesis method (Figures S1a and S2). Figure 1a shows the lattice fringes of the Pd NCs with an interplanar *d*-spacing of 1.94 Å, which corresponds to the (200) plane of *fcc* Pd [29]. The subsequent deposition of Bi on the Pd NCs was accomplished by reducing Bi^3+^ ions to Bi using NH_3_·BH_3_ as the reductant and Pd NCs as the seeds (Figure S1b). The inductively coupled plasma spectrometry (ICP-OES) results indicate that the actual Bi-to-Pd molar ratio is consistent with the feeding ratio (Table S1). According to the literature, the newly formed atoms should preferentially grow along the corners and edges of the pre-deposited metal, followed by the location of the facets to minimize the surface free energy [22,30]. 

Notably, the reducibility of the reductants is also vital for the selective deposition of Bi on Pd NCs. When the NaBH_4_ with strong reducibility was used as the reductant, some Bi were dispersed as nanoparticles instead of deposition on Pd NCs (Figure S3). In contrast, when weakening the reducibility of NH_3_·BH_3_ with NaOH (NH_3_·BH_3_-NaOH), the surface of the Pd NCs was coated by an amorphous phase, and selective deposition was not observed (Figure S4). According to the theory proposed by Xia, the growth pathway of atoms on seed has a strong dependence on the ratio between the rates for atom deposition and surface diffusion (i.e., *V*_deposition_/*V*_diffusion_), where *V*_diffusion_ is a thermodynamic parameter and *V*_deposition_ is related to the reducibility of the reductant [30]. Given that the samples were prepared at room temperature, the low value of *V*_diffusion_ allowed for the effect of Bi surface diffusion to be ignored. Consequently, the selective deposition of Bi at various locations on the Pd NCs could be controlled by changing the Bi-to-Pd feeding ratio and selecting the appropriate reductant.

The morphology of the Pd NCs@Bi was characterized by high-resolution transmission electron microscopy (HRTEM). As is shown in Figure 1b, the bulge-like structures (red dashed circles in Figure S5a–c) appear at the corners of the Pd NCs as the Bi-to-Pd molar ratio is 0.06. The line-scanning analysis results show that only Pd signals are detected at the locations of the Pd NCs, whereas strong Bi signals appear near the bulge-like structures (Figure S5d), indicating that Bi is significantly dispersed at the corners of Pd NCs. By further increasing the Bi-to-Pd molar ratio, it is evident that the bulge-like structures for Pd NCs@Bi_0.25_ are present at all the corners of the Pd NCs (Figure 1c and Figure S6a–c). The line-scanning images reveal that Bi is predominantly distributed at the corners of the Pd NCs (Figure S6d). As shown in the HRTEM images of Pd NCs@Bi_0.5_, the bulge-like structures are clearly visible at both the corners and the edge of the Pd NCs as the Bi-to-Pd molar ratio is 0.5 (Figure 1d and Figure S7a–c). The line-scanning analysis results further demonstrate that Bi is primarily distributed at the corners and edges of the Pd NCs (Figure S7d). Furthermore, the HRTEM images of Pd NCs@Bi_1_ show that the surface of the PdNCs is entirely covered by Bi when the Bi-to-Pd molar ratio is 1 (Figure 1e and Figure S8a–c). It is further proven through line-scanning analysis that Bi completely covers the Pd NCs, forming core-shell-like structures (Figure S8d). These results clearly prove that the precise deposition of Bi on the Pd NCs in the order of the corners, edges and facets can be achieved by adjusting the Bi concentration.

**Figure 2:**
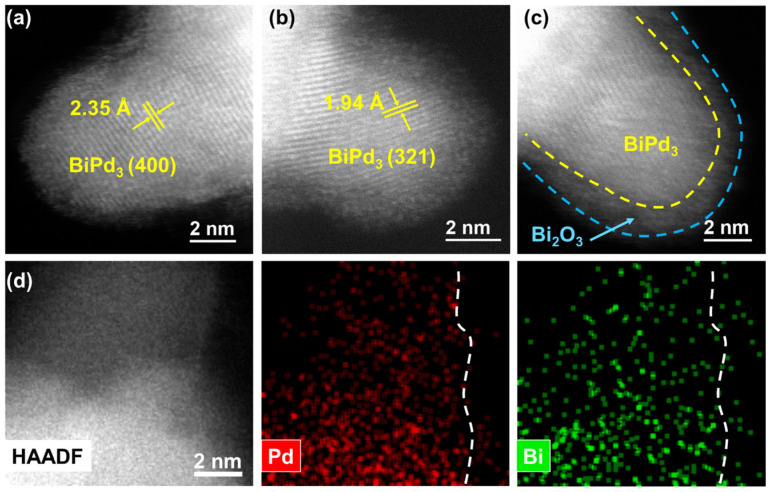
Microstructure of Pd NCs@Bi_0.5_. (**a**–**c**) HAADF-STEM images, (**d**) High-resolution HAADF image and elemental mapping analysis.

To further clarify the microstructures of Pd NCs@Bi, spherical aberration-corrected scanning transmission electron microscopy (ac-STEM) was employed. As shown in Figure 2a,b, the bulge-like structures in Pd NCs@Bi_0.5_ show lattice distances of ca. 2.35 and 1.94 Å, which are attributed to the (400) and (321) planes of the BiPd_3_ structure (Pmma (51) space group, orthorhombic structure) [26,31]. According to the literature and recent studies by our group, we speculate that the formation of the BiPd_3_ structure is the result of the interdiffusion of Bi and Pd in the radial direction due to strong interaction [32,33]. More importantly, it can be seen more clearly that the BiPd_3_ structure is covered by an amorphous phase (Figure 2c). The element mapping confirms that Pd and Bi are evenly dispersed within the bulge-like structures, which is also consistent with the BiPd_3_ structure, and Bi is also observed on the amorphous phase outside the BiPd_3_ structure (Figure 2d). Meanwhile, the XPS analysis of *α*-Al_2_O_3_-supported Pd NCs@Bi_0.5_ (Pd NCs@Bi_0.5_/Al_2_O_3_) shows that Bi_2_O_3_ is the dominant structure (Figure S9), which implies that the amorphous phase is Bi_2_O_3_. This could be attributed to the easy oxidation of metal Bi in the air [34]. The results above demonstrate that the deposition of Bi on Pd NCs forms a BiPd_3_ structure, which is subsequently covered by Bi_2_O_3_. Hence, the site-specific deposition of Bi can be considered as the selective coverage of Bi_2_O_3_ at the predetermined position of the Pd NCs. Additionally, the site-specific deposition of Bi can also be achieved on Pd NCs with a 7 nm size (Figure S10). As displayed in the HRTEM images of Pd_7nm_ NCs@Bi_0.5_, the bulge-like structures are found at the corners of the 7 nm sized Pd NCs, which is further confirmed by the line-scanning profile (Figure S11).

### 2.2. Catalytic Performance in Acetylene Hydrogenation

The selective deposition of Bi on Pd NCs provides the foundation for an in-depth comprehension of the structure-activity relationship of Pd catalysts in acetylene-selective hydrogenation reactions. The Pd NCs@Bi/Al_2_O_3_ catalysts were synthesized by a conventional sol immobilization method [35] using *α*-Al_2_O_3_ as the support. The actual content of Pd in the Pd NCs@Bi/Al_2_O_3_ catalysts is approximately 0.2 wt%, determined by ICP-OES (Table S2). Due to the low loading of Pd and Bi in the catalysts, only Al_2_O_3_ diffraction peaks were observed in the corresponding XRD patterns (Figure S12). The catalytic performances of Pd NCs@Bi/Al_2_O_3_ in the acetylene hydrogenation were evaluated under the excess ethylene atmosphere, simulating the front-end conditions. 

**Figure 3:**
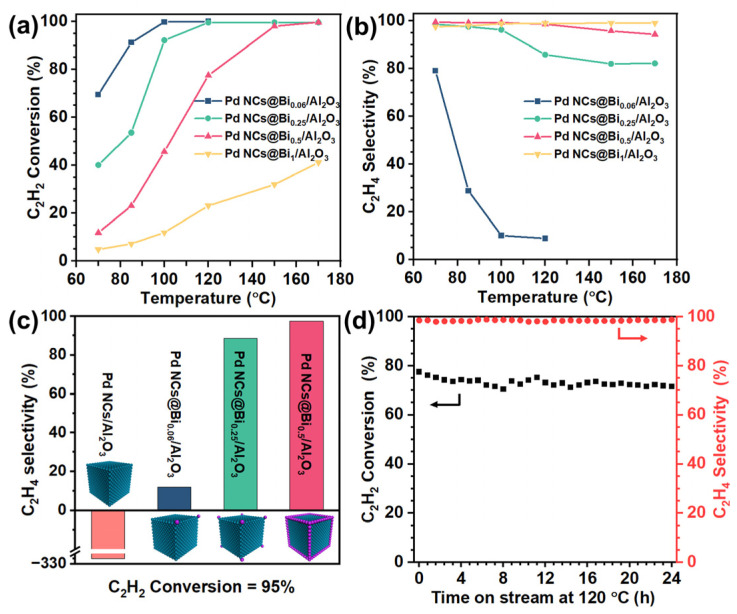
Catalytic performances of Pd NCs@Bi/Al_2_O_3_ in acetylene hydrogenation. (**a**) C_2_H_2_ conversion and (**b**) selectivity to C_2_H_4_, (**c**) the selectivity to C_2_H_4_ for 95% C_2_H_2_ conversion over different catalysts, (**d**) long−term stability test of Pd NCs@Bi_0.5_/Al_2_O_3_ at 120 °C. (Reaction conditions: space velocity of 120,000 mL h^−1^ g^−1^ and reactant gases: 1.0 vol % C_2_H_2_, 20.0 vol % C_2_H_4_, 20.0 vol % H_2_ and 59.0 vol % N_2_).

Consistent with the literature results, the Pd NCs/Al_2_O_3_ catalyst exhibits 100% C_2_H_2_ conversion and −315% C_2_H_4_ selectivity at 70 °C, indicating an uncontrollable over-hydrogenation capability of the Pd catalysts, accompanied by temperature runaway [36]. The Bi/Al_2_O_3_ catalyst shows no catalytic activity for acetylene hydrogenation, which is consistent with our previous report [36]. This means that the precise coverage of Bi on the Pd NCs is the selective poisoning to the active site of Pd NCs [37]. Therefore, increasing the Bi-to-Pd molar ratio could improve the poisoning degree. Herein, the degree of Bi deposition is defined as the poisoning degree. Interestingly, the catalytic performance varied significantly when the poisoning degree changed. As shown in Figure 3a,b, the Pd NCs@Bi_0.06_/Al_2_O_3_ catalyst with poisoning on the corners of the Pd NCs shows 10.1% C_2_H_4_ selectivity at 100% C_2_H_2_ conversion, which infers a fundamental improvement compared to the catalytic performance of the Pd NCs/Al_2_O_3_ catalyst. Importantly, as the poisoning degree increases, the C_2_H_2_ conversion gradually decreases, and the C_2_H_4_ selectivity increases at the same reaction temperature, which exhibits the typical trade-off between high catalytic activity and selectivity. Figure 3c compares the C_2_H_4_ selectivity of all catalysts at the 95% C_2_H_2_ conversion. The C_2_H_4_ selectivity continues to increase with the increasing poisoning degree. Specifically, when the corners and edges of the Pd NCs are poisoned, the Pd NCs@Bi_0.5_/Al_2_O_3_ catalyst achieves the best catalytic performance, namely 99.7% C_2_H_2_ conversion and 94.3% C_2_H_4_ selectivity at 170 °C. This result confirms that moderately poisoning the corners and edges while preserving the facet sites of Pd NCs is responsible for the selective acetylene hydrogenation. It is noteworthy that the Pd NCs@Bi_1_/Al_2_O_3_ catalyst exhibits only 41.2% C_2_H_2_ conversion, even at 170 °C, due to an excessive poisoning extent. The above results prove that the poisoning-specific sites of Pd NCs can effectively regulate the catalytic performance to achieve the optimal trade-off between high conversion and selectivity. Furthermore, the long-term stability of the Pd NCs@Bi_0.5_/Al_2_O_3_ catalyst was evaluated. As depicted in Figure 3d, the C_2_H_4_ selectivity remains constant at 98% over 24 h, and C_2_H_2_ conversion also remains almost steady, decreasing slightly from 76% to 71% within the first 6 h. The HRTEM images of the spent Pd NCs@Bi_0.5_/Al_2_O_3_ catalyst show that the bulge-like structures are maintained (Figure S13), indicating that the stable structure formed by the selective coverage of Bi on the Pd NCs accounts for excellent long-term stability.

### 2.3. Study on Reaction Mechanism

**Figure 4:**
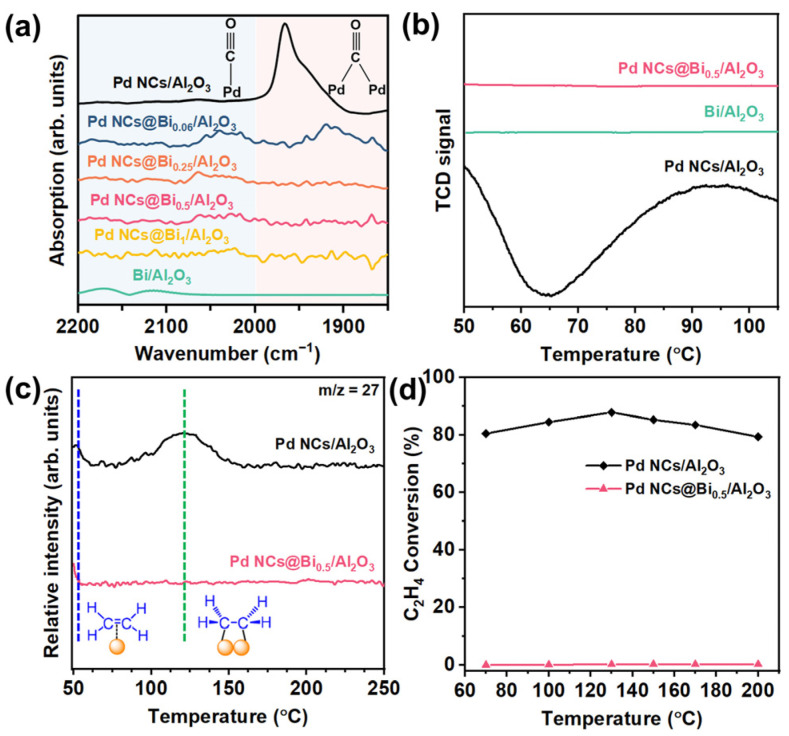
Study on reaction mechanism. (**a**) Infrared spectroscopy of CO adsorption, (**b**) H_2_−TPR profiles of Pd NCs/Al_2_O_3_, Bi/Al_2_O_3_ and Pd NCs@Bi_0.5_/Al_2_O_3_ catalysts, (**c**) C_2_H_4_−TPD profiles of Pd NCs/Al_2_O_3_ and Pd NCs@Bi_0.5_/Al_2_O_3_ catalysts, (**d**) C_2_H_4_ conversion as a function of reaction temperature. (Reaction conditions: space velocity of 120,000 mL h^−1^ g^−1^ and reactant gases: 1.0 vol % C_2_H_4_, 20.0 vol % H_2_ and 79 vol % N_2_).

To investigate the reaction mechanism and gain insight into the relationship between the selectively poisoned active sites and catalytic performance, the in situ diffuse reflectance infrared Fourier transform spectroscopy (DRIFTS) was performed using CO as the probe molecule. As shown in Figure 4a, the peaks in the range 2200−2000 cm^−1^ could be attributed to the adsorption of linear-bonded CO. The signal peak in the range 2000−1850 cm^−1^ could be assigned to the adsorption of bridge-bonded CO [38]. Clearly, the signal of the adsorption of bridge-bonded CO for the Pd NCs@Bi_0.06_/Al_2_O_3_ catalyst is decreased compared to that of Pd NCs/Al_2_O_3_. When the corner and edge sites of the Pd NCs were poisoned, no obvious signals belonging to the adsorption of bridge-bonded CO were observed for the Pd NCs Bi_0.5_/Al_2_O_3_ catalyst. These results suggest that the coordination environment of Pd can be effectively regulated by varying the poisoning degree [24]. 

Theoretical calculations have shown that ideal acetylene semi-hydrogenation catalysts should have moderate hydrogen dissociation and weak ethylene adsorption ability [39]. Strong hydrogen dissociation ability for Pd catalysts tends to generate excess active hydrogen atoms, which migrate to the subsurface region of Pd to form the *β*-PdH*_x_* [40]. The *β*-PdH*_x_* is considered the active species for over-hydrogenation by forming the high coordination Pd sites [41]. As shown in Figure 4b, the H_2_-TPR test shows the characteristic peak of *β*-PdH*_x_* at around 65°C for the Pd NCs/Al_2_O_3_ catalyst, while for the Pd NCs@Bi_0.5_/Al_2_O_3_ catalyst, such a peak is not observed. The results of DRIFTS and H_2_-TPR suggest that selectively poisoning the corner and edges of Pd NCs decreases the Pd–Pd coordination number and weakens the hydrogen dissociation ability, thereby inhibiting the formation of the *β*-PdH*_x_*. Note that the characteristic peak of *β*-PdH*_x_* is also not observed for Bi/Al_2_O_3_, likely due to the absence of a hydrogen dissociation capability. 

The C_2_H_4_-TPD characterization can be used to further investigate the selective poisoning on the ethylene adsorption/desorption behaviors. Figure 4c shows the characteristic peaks at about 50 °C and 120 °C for the Pd NCs/Al_2_O_3_ catalyst. The peak at approximately 50 °C could be attributed to the *π*-bonded ethylene, which is weakly adsorbed and benefits the desorption of ethylene. The peak centered around 120 °C represents the strong di-*σ*-bonded ethylene, which is unfavorable for desorption and results in the over-hydrogenation of ethylene into ethane [34,42]. For the Pd NCs@Bi_0.5_/Al_2_O_3_ catalyst, the peak of di-*σ*-bonded ethylene is not observed, and only the characteristic peak of *π*-bonded ethylene remains. This suggests that it is hard to over-hydrogenate ethylene to ethane over the Pd NCs@Bi_0.5_/Al_2_O_3_ catalyst, which is consistent with the result of the catalytic performances (Figure 3a,b). This result is further confirmed by the ethylene hydrogenation experiment (Figure 4d). Due to the weak adsorption of ethylene, the Pd NCs@Bi_0.5_/Al_2_O_3_ catalyst exhibits lower than 5% C_2_H_4_ conversion, whereas the C_2_H_4_ conversion for Pd NCs/Al_2_O_3_ is over 80% in the whole test temperature. These results demonstrate that by precisely controlling the deposition of Bi on specific locations of the Pd NCs to poison the corner and edge sites, a moderate hydrogen dissociation capacity and weak ethylene adsorption could be obtained to achieve the optimal compromise between high catalytic activity and selectivity. 

The selective deposition of Bi on Pd NCs has be accomplished with 7 nm sized Pd NCs (Figure S11). Accordingly, it is investigated whether the site-specific deposition of Bi on Pd_7nm_ NCs can regulate the catalytic performance of Pd_7nm_ NCs@Bi/Al_2_O_3_ catalysts. The Pd_7nm_ NCs@Bi/Al_2_O_3_ catalysts were synthesized using the same method as the Pd NCs@Bi/Al_2_O_3_ catalysts, with 0.2 wt% Pd content, measured by ICP-OES (Table S2). Figure S14 shows that as the degree of the Bi deposition increases, the C_2_H_2_ conversion decreases and the C_2_H_4_ selectivity increases at the same reaction temperature, which is consistent with the results of Pd NCs@Bi/Al_2_O_3_. Notably, to achieve the best catalytic performance of Pd_7nm_ NCs@Bi/Al_2_O_3_, a higher Bi-to-Pd molar ratio compared to Pd NCs@Bi/Al_2_O_3_ is required. This may be attributed to the larger specific surface area of the smaller Pd NCs.

The Pd_7nm_ NCs@Bi_1_/Al_2_O_3_ catalyst exhibits an excellent catalytic performance, with 97.8% C_2_H_4_ selectivity at 98.2% C_2_H_2_ conversion. These results suggest that the effect of the site-specific deposition of Bi on the catalytic performance can be applied to Pd NCs of both 20 nm and 7 nm dimensions.

These results can provide insight into the relationship between active sites and catalytic performance and, more importantly, provide ideas for designing catalysts for industrial production. The selective deposition of Bi on Pd NCs of various sizes raises the question of whether it can also be applied to Pd nanoparticles (Pd NPs) with irregular morphology. To this end, a facile stepwise reduction method was proposed to synthesize the selectively Bi-deposited Pd nanoparticle catalysts (Pd NPs@Bi-based catalysts, Figure S15). According to the ICP-OES analysis, the catalysts contain 0.2 wt% Pd (Table S2). Surprisingly, the Pd NPs@Bi-based catalysts showed an outstanding catalytic performance: 96.4% C_2_H_4_ selectivity at 99.9% C_2_H_2_ conversion for Pd NPs@Bi_1_/Al_2_O_3_ and 93.8% C_2_H_4_ selectivity at 99.7% C_2_H_2_ conversion for Pd NPs@Bi_0.8_/CaCO_3_ (Figure S16). Moreover, these results also imply that the support could be more irrelevant to the catalytic performance, which facilitates the screening of suitable support for industrial catalysts. 

## 3. Materials and Methods

### 3.1. Materials

Polyvinylpyrrolidone (K29-32, MW = 58,000, ≥ 99%, Aladdin Chemicals, Shanghai, China), KBr (ACS, ≥ 99%, Aladdin Chemicals, Shanghai, China), K_2_PdCl_4_ (99.99% metals basis, Aladdin Chemicals, Shanghai, China), ascorbic acid (ACS, ≥ 99%, Aladdin Chemicals, Shanghai, China), KOAc (99.0%, Aladdin Chemicals, Shanghai, China), ethylene glycol (99%, Sinopharm Chemicals, shanghai, China), Bi(NO_3_)_3_·5H_2_O (99.9%, Aladdin Chemicals, Shanghai, China), borane-ammonia complex (NH_3_·BH_3_, 97%, Aladdin Chemicals, Shanghai, China), *α*-Al_2_O_3_ (99.9%, Aladdin Chemicals, Shanghai, China), CaCO_3_ (99.9%, Aladdin Chemicals, Shanghai, China) were used as received without further purification.

### 3.2. Synthesis of Pd NCs

The Pd NCs with different sizes were prepared by the liquid-phase synthesis method illustrated in Figure S1a. Typically, 67 mg of polyvinylpyrrolidone (PVP), 36 mg of ascorbic acid (AA) and 360 mg of KBr were dispersed in 5 mL of aqueous solution. Then, 1 mL of 120 mM K_2_PdCl_4_ aqueous solution was added, and the mixture was stirred for 3 h. After cooling to room temperature, the 20 nm sized Pd NCs were obtained by centrifugation and washing with water and acetone for several times to remove the residual PVP. The 7 nm sized Pd nanocubes (Pd_7 nm_ NCs) were prepared by adding 0.12 mmol of KOAc [29], following the same procedures as the 20 nm sized Pd NCs sample. 

### 3.3. Synthesis of Pd NCs@Bi and Pd NCs@Bi/Al_2_O_3_

Pd NCs@Bi*_x_* (*x* is the Bi-to-Pd molar ratio) were synthesized by a seed growth method, illustrated in Figure S1b. A certain amount of Pd NCs aqueous solution was dissolved in 4 mL of ethylene glycol (EG), followed by adding a certain amount of Bi(NO_3_)_3_·5H_2_O dissolved in EG. The mixture was stirred for 1 h before 1 mL of 0.5 M borane-ammonia complex (NH_3_·BH_3_) was added in a dropwise manner. The Pd NCs@Bi samples were obtained by centrifugation and washing with water and acetone several times. 

The Pd NCs@Bi/Al_2_O_3_ catalysts were synthesized by a conventional sol immobilization method [35]. Typically, 200 mg of *α*-Al_2_O_3_ was added into a certain amount of Pd NCs@Bi aqueous solution for a nominal Pd loading of 0.2 wt%. The mixture was stirred for 2 h at room temperature before centrifugation and washing several times with water and acetone, and subsequently oven-dried overnight at 60 °C. 

### 3.4. Synthesis of Pd NPs@Bi/Al_2_O_3_ and Pd NPs@Bi/CaCO_3_

The Pd NPs@Bi/Al_2_O_3_ and Pd NPs@Bi/CaCO_3_ catalysts were prepared by stepwise reduction method. Typically, 200 mg of *α*-Al_2_O_3_ or CaCO_3_ was added into K_2_PdCl_4_ aqueous solution and 5 mL of acetone to ensure a nominal Pd loading of 0.2 wt%. The mixture was stirred for 1 h after 1 mL of 0.5 M borane-ammonia complex (NH_3_·BH_3_) added in a dropwise manner. The calculated amount of Bi(NO_3_)_3_·5H_2_O dissolved in EG solution was added dropwise into the mixture. The suspension continued to be stirred for another 1 h, followed by centrifugation and washing with water and acetone, and subsequently oven-dried overnight at 60 °C. 

### 3.5. Characterization

The actual metal contents of all the samples were quantified by inductively coupled plasma spectrometry (ICP-OES, PerkinElmer 8300, PerkinElmer CORP, Waltham, MA, USA). High-resolution transmission electron microscopy (HRTEM) images and line-scanning analysis data were recorded on a field emission transmission electron microscope JEM2100F equipped with an energy-dispersive X-ray spectroscope (EDS). High-angle annular dark-field scanning transmission electron microscopy (HAADF-STEM) images and elemental mapping data were obtained by the Cs-corrected STEM (Titan G2 80-200 ChemiSTEM, FEI COPR, Portland, OR, USA) equipped with an EDS. The X-ray diffraction (XRD) analysis was performed on a Bruker D_2_-Phaser diffractometer operating at a scanning step of 2°/min with a Cu Kα radiation source. The X-ray photoelectron spectroscopy (XPS) was performed using a VG Scientific ESCALAB Mark II spectrometer. The in situ diffuse reflectance infrared Fourier transform spectroscopy (DRIFTS) test over catalysts was characterized by Nicolet iS50 instrument. Prior to the test, the catalysts were pretreated in H_2_/Ar at 200 °C for 1 h and then treated with Ar for 30 min at room temperature. The spectrum collected at this time was used as the background spectrum. After treating the catalysts with CO for 30 min and then purging them with Ar for 30 min, the DRIFTS spectra were recorded until there was no change in the peak intensity. Temperature-programmed reduction (TPR) experiments were carried out on Micromeritics ChemiSorb 2920 automatic chemisorption analysis instrument (Micromeritics Instrument CORP, Norcross, GA, USA). The catalysts were dried at 100 °C and then pre-reduced in 10 vol % H_2_/Ar at 200 °C for 1 h, followed by cooling down to room temperature under Ar. The TPR data were collected under 10 vol % H_2_/Ar for hydrogen adsorption, followed by heating catalysts from room temperature to 200 °C at 10 °C/min under Ar. The temperature programmed desorption of C_2_H_4_ (C_2_H_4_-TPD) experiments were performed on a Microtrac BELCat II instrument (MicrotracBEL CORP, Tokyo, Japan). The catalysts were pretreated in 10 vol % H_2_/Ar at 200 °C for 1 h and then purged with He (30 mL/min) to remove the physically adsorbed C_2_H_4_ after adsorption with 20 vol % C_2_H_4_/He at room temperature. The catalysts were heated to 300 °C at a rate of 10 °C/min, followed by detecting the products of the desorption process on VG Sensorlab mass spectrometer.

### 3.6. Catalytic Tests

Acetylene hydrogenation tests were performed in a fixed-bed reactor with 8 mm inner diameter quartz tubes at atmospheric pressure. The catalyst evaluation conditions simulated the reaction conditions of industrial front-end hydrogenation under ethylene-rich conditions with reaction gases of 1.0 vol % C_2_H_2_, 20.0 vol % C_2_H_4_, 20.0 vol % H_2_ and 59.0 vol % N_2_. The catalyst (30 mg) was mixed with 20–40 mesh quartz sand (400 mg) and then placed in a quartz reactor. A thermocouple sleeve with an external diameter of 6 mm was inserted into the quartz reactor to detect the real-time temperature of the catalyst bed during the reaction. Therefore, the temperature in the catalytic reaction test represents the real-time temperature of the catalyst bed. The as-prepared catalysts were treated in 10 vol % H_2_/Ar at 200 °C for 1 h prior to catalytic experiments. The total flow rate of the reaction gas was set to 60 mL/min, with a space velocity of 120,000 mL h^−1^ g^−1^, and was then pumped into the quartz reactor for on-line detection. The gas products were analyzed by the gas chromatography equipped with an FID detector. Note that the carbon balance was >99% and oligomers production is negligible due to the hydrogen-rich atmosphere and short contact time. The C_2_H_2_ conversion and C_2_H_4_ selectivity were calculated as:

(1) $$C_{2}H_{2}\;\mathrm{conversion}=\frac{{{[C}_{2}H_{2}]}_{\mathrm{inlet}}-{{[C}_{2}H_{2}]}_{\mathrm{outlet}}}{{{[C}_{2}H_{2}]}_{\mathrm{inlet}}}\times 100\%$$

(2) $$C_{2}H_{4}\;\mathrm{selectivity}=\frac{{{[C}_{2}H_{2}]}_{\mathrm{inlet}}-{{[C}_{2}H_{2}]}_{\mathrm{outlet}}-{{[C}_{2}H_{6}]}_{\mathrm{outlet}}}{{{[C}_{2}H_{2}]}_{\mathrm{inlet}}-{{[C}_{2}H_{2}]}_{\mathrm{outlet}}}\times 100\%$$

## 4. Conclusions

In summary, the selective deposition of Bi on Pd NCs has been achieved to allow Bi_2_O_3_ cover in the priority order from the corners to edges and then to the facets of Pd NCs. Benefitting from the moderate hydrogen dissociation against the formation of hydride and the weak ethylene adsorption, the supported Pd NCs@Bi catalyst with covered corners and edges exhibits 94.3% C_2_H_4_ selectivity at 99.7% C_2_H_2_ conversion and good long–term stability for acetylene hydrogenation. By broadening the research perspectives, the selectively Bi-deposited Pd nanoparticle catalysts prepared by a facile stepwise reduction method show >93% C_2_H_4_ selectivity at >99% C_2_H_2_ conversion. This design strategy offers a distinctive perspective in the precise modulation of the active sites for the rational design of highly selective catalysts.

## Data Availability

The data that support the findings of this study are available in the Supplementary Materials of this article or from the corresponding author upon reasonable request.

## Supplementary Materials

The following supporting information can be downloaded at: https://www.mdpi.com/article/10.3390/molecules28052335/s1. Table S1: ICP-OES analysis of Pd NCs@Bi*_x_*; Table S2: ICP-OES analysis of catalysts. Figure S1: Schematic illustration of Pd NCs and Pd NCs@Bi synthesis. Figure S2: HRTEM image and size distribution of 20 nm sized Pd NCs. Figure S3: HRTEM image and EDS elemental maps of Pd NCs@Bi_0.25_ using NaBH_4_ as reductant. Figure S4: HRTEM images of Pd NCs@Bi_0.25_ using NH_3_·BH_3_-NaOH as reductant. Figures S5–S8: HRTEM images and line scanning profile of Pd NCs@Bi*_x_*. Figure S9: Bi 4*f* XPS spectra of Pd NCs@Bi_0.5_/Al_2_O_3_ catalyst. Figure S10: HRTEM image and size distribution of 7 nm sized Pd NCs. Figure S11: HRTEM images and line scanning profile of Pd_7nm_ NCs@Bi_0.5_. Figure S12: XRD patterns of Pd NCs/Al_2_O_3_ and Pd NCs@Bi*_x_*/Al_2_O_3_ catalysts. Figure S13: HRTEM images of Pd NCs@Bi_0.5_/Al_2_O_3_-spent catalyst. Figure S14: Catalytic performances of Pd_7nm_ NCs@Bi*_x_*/Al_2_O_3_ catalysts in acetylene hydrogenation. Figure S15: Schematic illustration of Pd NPs@Bi-based catalysts synthesis. Figure S16: Catalytic performances of Pd NPs@Bi based catalysts in acetylene hydrogenation.

## Sample Availability

Samples of the compounds Pd NCs@Bi/Al_2_O_3_ are available from the authors.
