# Supplementary material for: Controllable Deposition of Bi onto Pd for Selective Hydrogenation of Acetylene
Source: Molecules. 2023 Mar 3;28(5):2335. doi: 10.3390/molecules28052335 (PMC10005703; doi:10.3390/molecules28052335)
Supplement: Supplementary file 1 [file molecules-28-02335-s001.zip › molecules-2242164-supplementary.pdf]

Supporting Information

Controllable deposition of Bi onto Pd for selective hydrogenation of acetylene

Hongquan Kang[1,†], Baohui Lou[1,†], Yue Wang[1], Yilin Zhao[1], Juanjuan Liu[2], Jianzhou Wu[1,*], Shihui Zou[1,*] and Jie Fan[1,3*]

1. Key Lab of Applied Chemistry of Zhejiang Province, Department of Chemistry, Zhejiang University, 310027 Hangzhou, China.

2. College of Materials & Environmental Engineering, Hangzhou Dianzi University, Hangzhou 310036, China

3. Shanxi-Zheda Institute of Advanced Materials and Chemical Engineering, Taiyuan, 030032, China

*Correspondences: jfan@zju.edu (J. Fan); xueshan199@163.com (S.-H. Zou); wjzclig@gmail.com (J.-Z. Wu)

†These authors contributed equally to this work.

Table S1. ICP-OES analysis of Pd NCs@Bi_x.

Samples	Nominal ratio of Bi/Pd (mol%)[a]	Actual ratio of Bi/Pd (mol%)[b]
Pd NCs@$Bi_{0.06}$	0.06	0.06
Pd NCs@$Bi_{0.25}$	0.25	0.26
Pd NCs@$Bi_{0.5}$	0.50	0.47
Pd NCs@Bi_1	1.00	1.02

[a] Determined by the concentration of PdNCs and Bi precursors added.

[b] Determined by ICP-OES analysis.

Table S2. ICP-OES analysis of catalysts.

Catalysts	Loading amount of Pd / wt%	Loading amount of Bi/ wt%
Pd NCs@$Bi_{0.06}$/Al_2O_3	0.23	0.03
Pd NCs@$Bi_{0.25}$/Al_2O_3	0.19	0.12
Pd NCs@$Bi_{0.5}$/Al_2O_3	0.21	0.21
Pd NCs@Bi_1/Al_2O_3	0.19	0.42
Pd_{7nm} NCs@$Bi_{0.5}$/Al_2O_3	0.18	0.20
Pd_{7nm} NCs@Bi_1/Al_2O_3	0.22	0.39
Pd_{7nm} NCs@$Bi_{1.3}$/Al_2O_3	0.19	0.50
Pd NPs@Bi_1/Al_2O_3	0.18	0.38
Pd NPs@$Bi_{0.8}$/$CaCO_3$	0.22	0.29

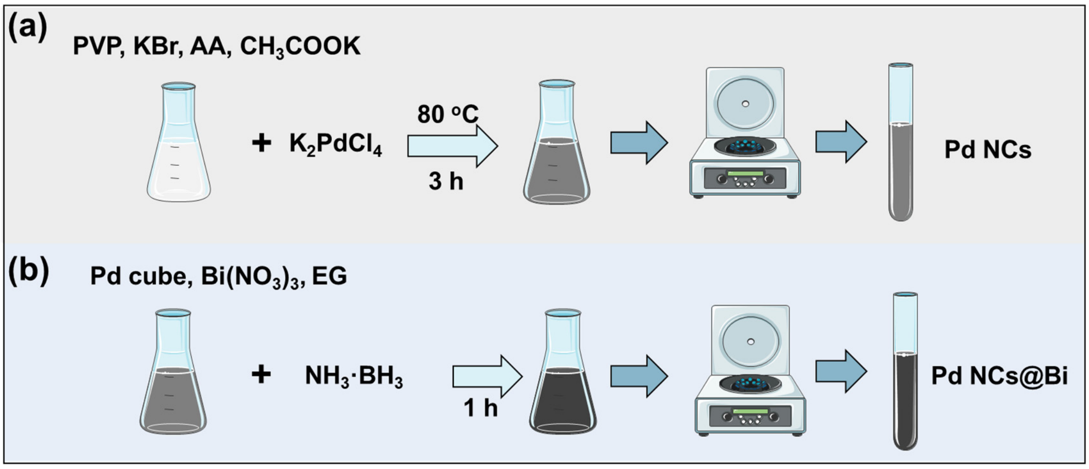

Figure S1. Schematic illustration of (a) Pd nanocubes (Pd NCs) and (b) site-specific deposition of Bi on Pd nanocubes (Pd NCs@Bi) synthesis.

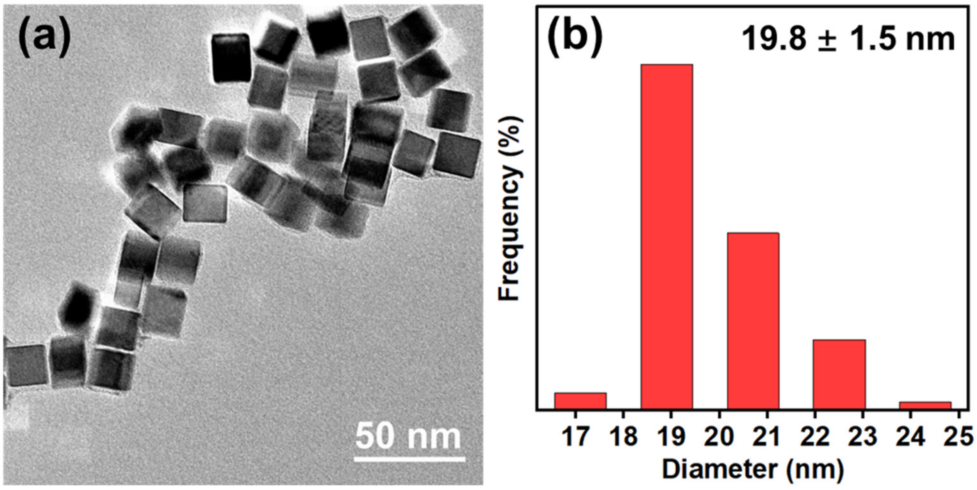

Figure S2. (a) HRTEM image and (b) size distribution of 20 nm sized Pd NCs.

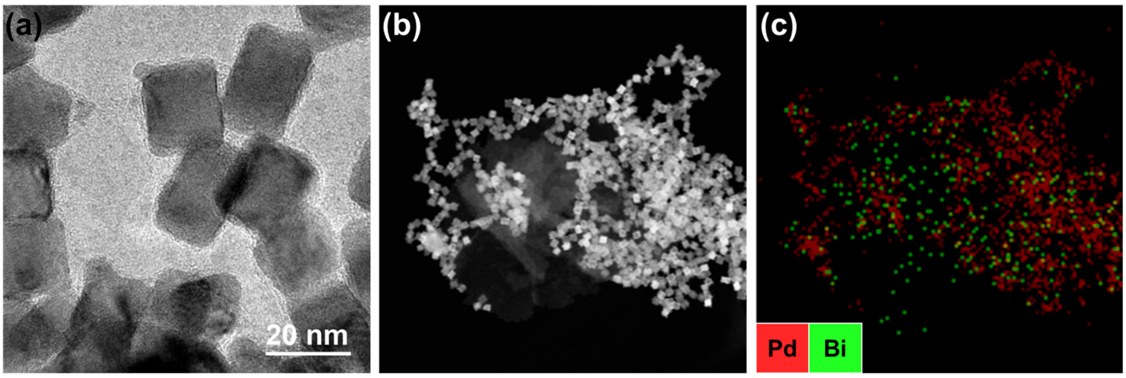

Figure S3. (a) HRTEM image and (b-c) EDS elemental maps of Pd NCs@$Bi_{0.25}$ using $NaBH_4$ as reductant.

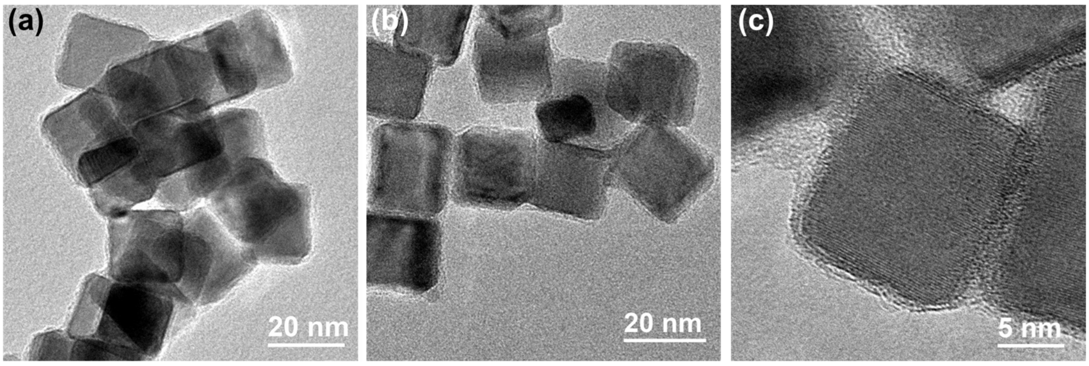

Figure S4. HRTEM images of Pd NCs@$Bi_{0.25}$ using $NH_3 \cdot BH_3$-NaOH as reductant.

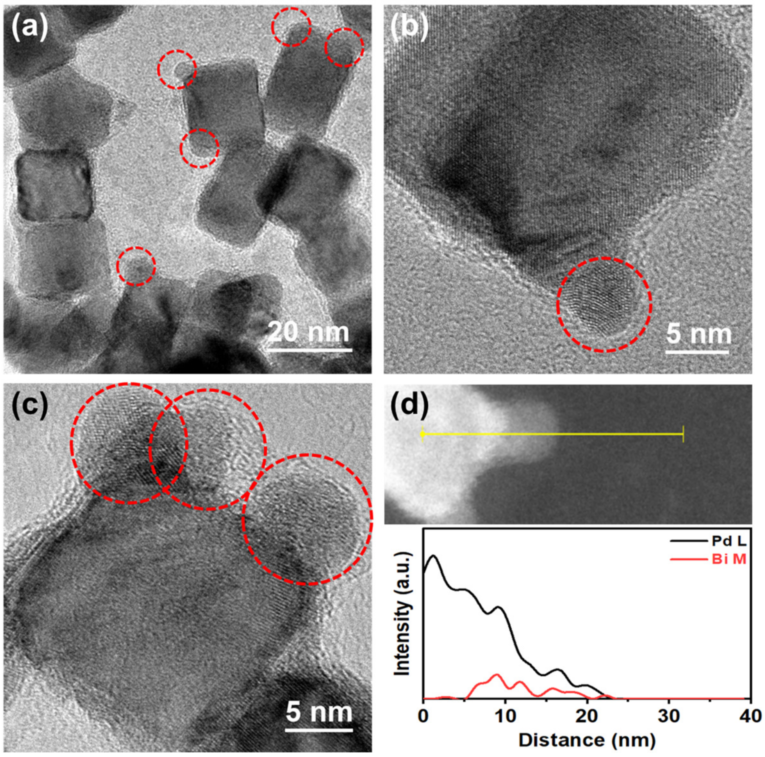

Figure S5. (a-c) HRTEM images and (d) line scanning profile of Pd NCs@$Bi_{0.06}$.

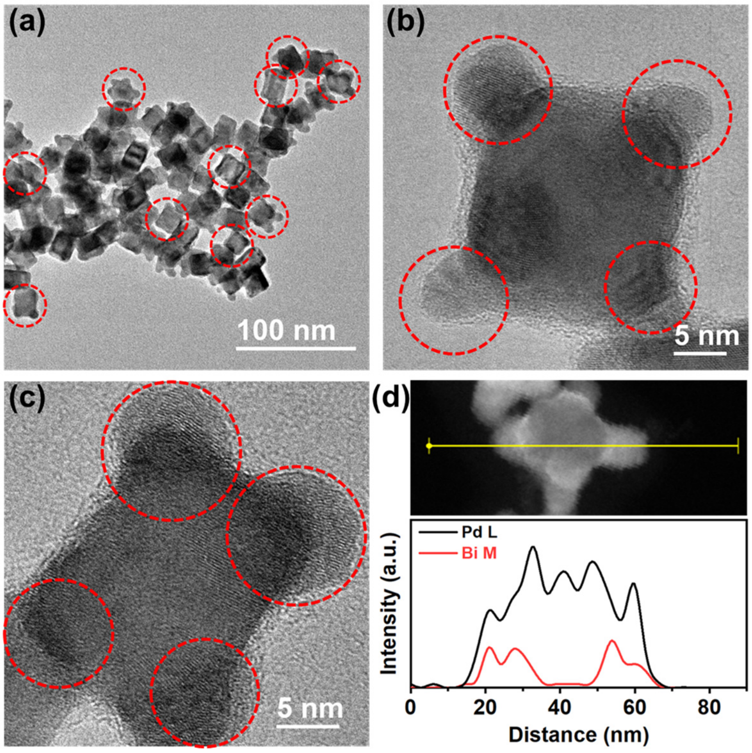

Figure S6. (a-c) HRTEM images and (d) line scanning profile of Pd NCs@$Bi_{0.25}$.

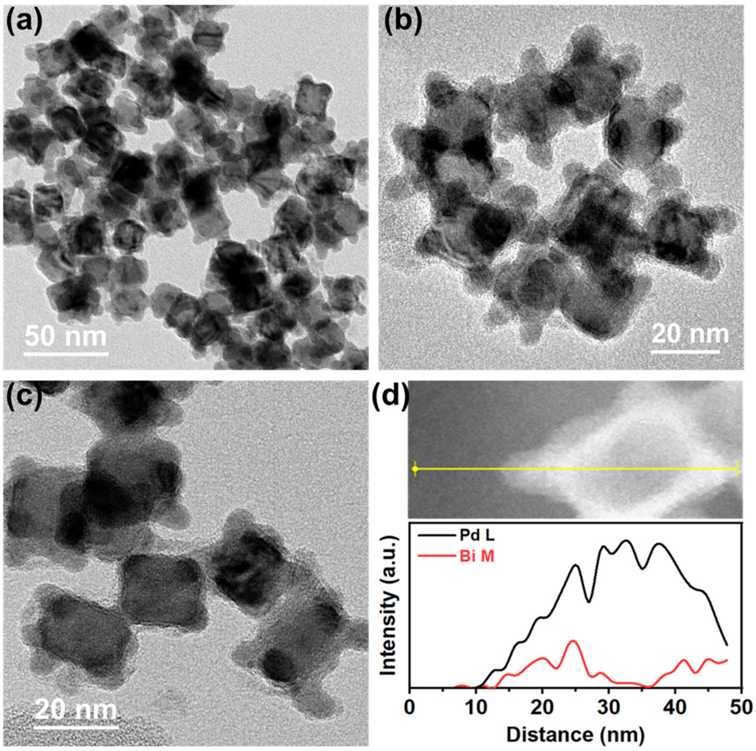

Figure S7. (a-c) HRTEM images and (d) line scanning profile of Pd NCs@$Bi_{0.5}$.

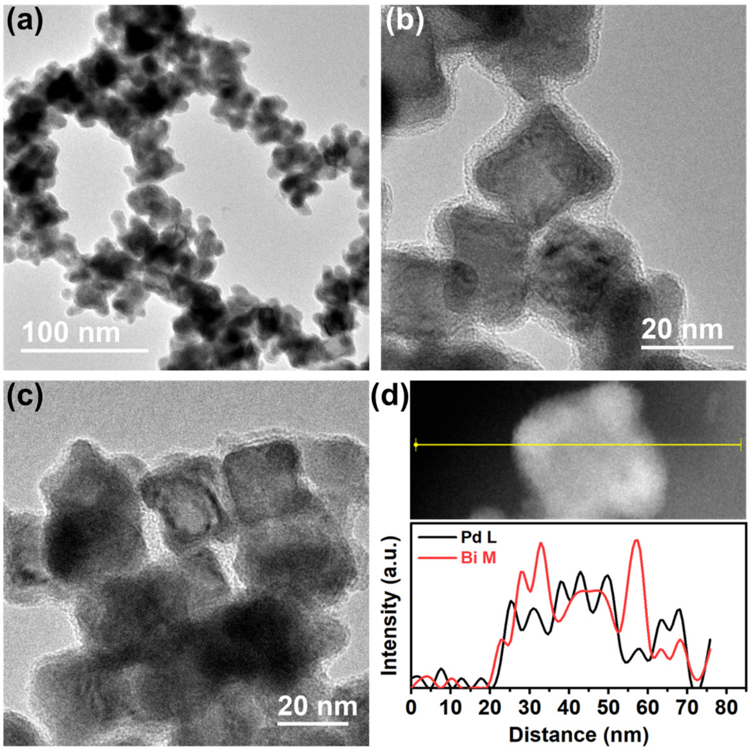

Figure S8. (a-c) HRTEM images and (d) line scanning profile of Pd NCs@Bi_1.

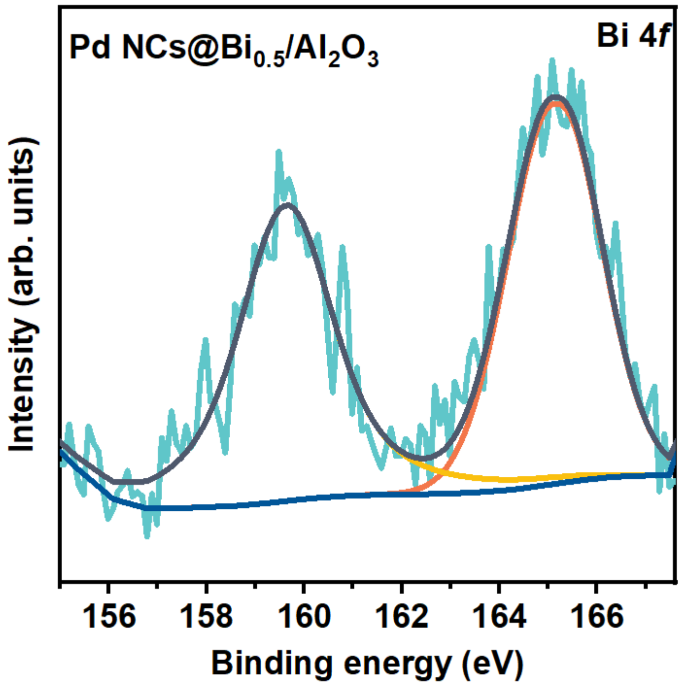

Figure S9. Bi 4*f* XPS spectra of Pd NCs@$Bi_{0.5}/Al_2O_3$ catalyst. Notably, Pd 3d signal was undetectable due to the low Pd content (0.2 wt.%) and the fact that Bi coating on the surface of the Pd NCs could affect the signal of Pd.

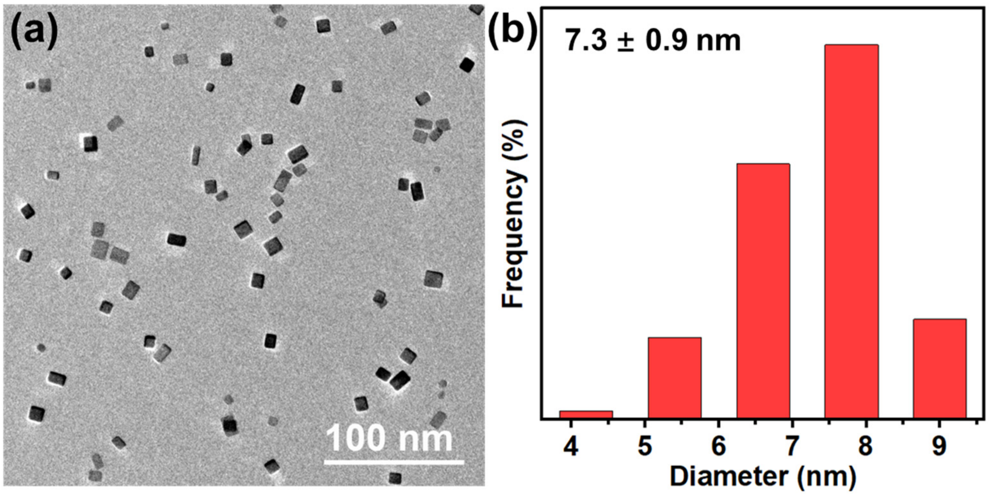

Figure S10. (a) HRTEM image and (b) size distribution of 7 nm sized Pd NCs.

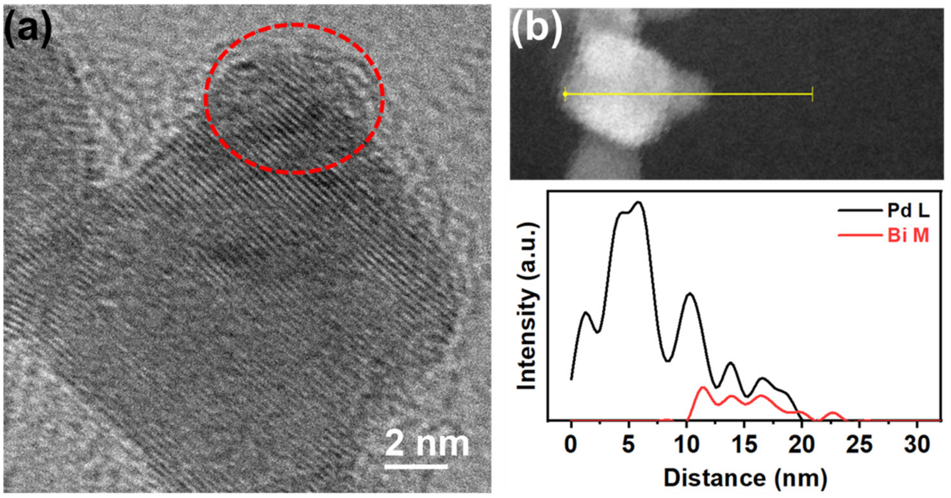

Figure S11. (a) HRTEM images and (b) line scanning profile of Pd_{7nm} NCs@$Bi_{0.5}$.

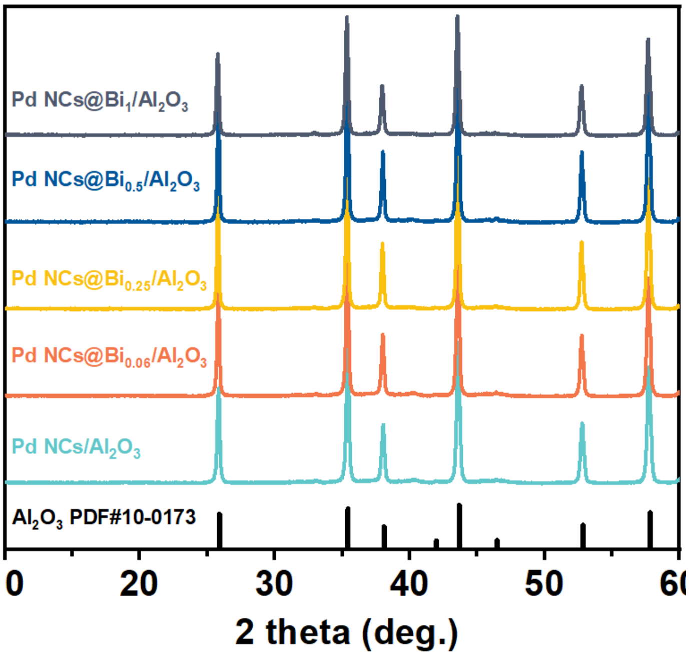

Figure S12. XRD patterns of Pd NCs/Al_2O_3, Pd NCs@$Bi_{0.06}$/Al_2O_3, Pd NCs@$Bi_{0.25}$/Al_2O_3, Pd NCs@$Bi_{0.5}$/Al_2O_3 and Pd NCs@Bi_1/Al_2O_3 catalysts.

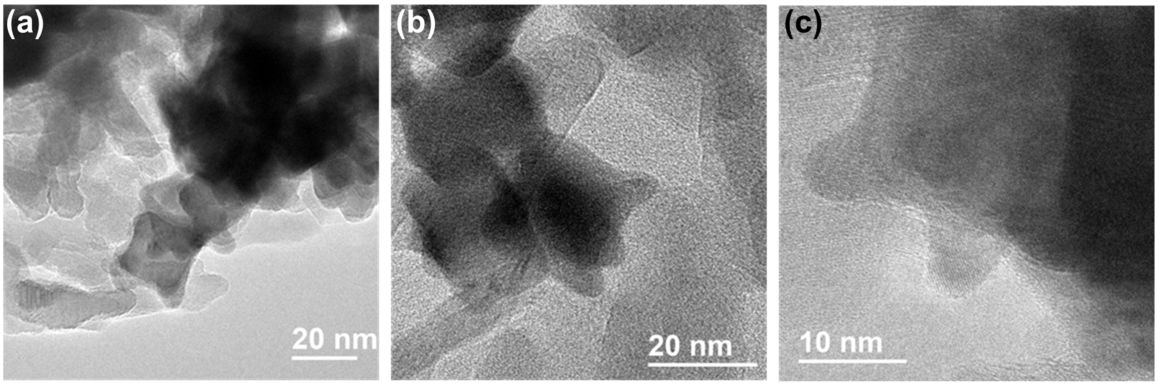

Figure S13. HRTEM images of Pd NCs@$Bi_{0.5}$/Al_2O_3-spent catalyst.

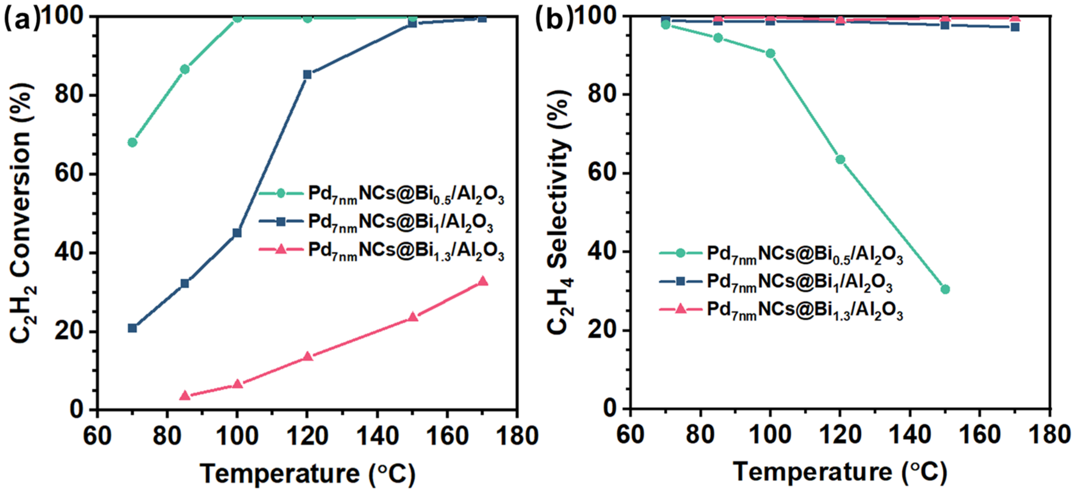

Figure S14 Catalytic performances of Pd_{7nm} NCs@Bi_x/Al_2O_3 catalysts in acetylene hydrogenation. (a) C_2H_2 conversion as a function of reaction temperature and (b) C_2H_4 selectivity as a function of reaction temperature. (Reaction conditions: space velocity of 120000 mL h^{-1} g^{-1} and reactant gases: 1.0 vol % C_2H_2, 20.0 vol % C_2H_4, 20.0 vol % H_2, and 59 vol % N_2).

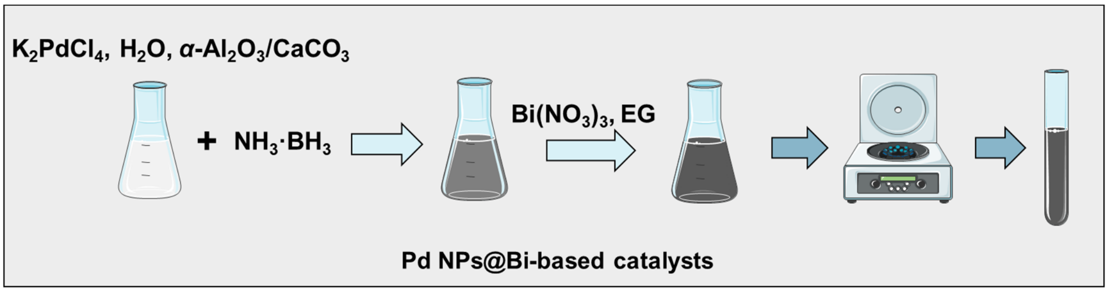

Figure S15. Schematic illustration of Pd NPs@Bi-based catalysts synthesis.

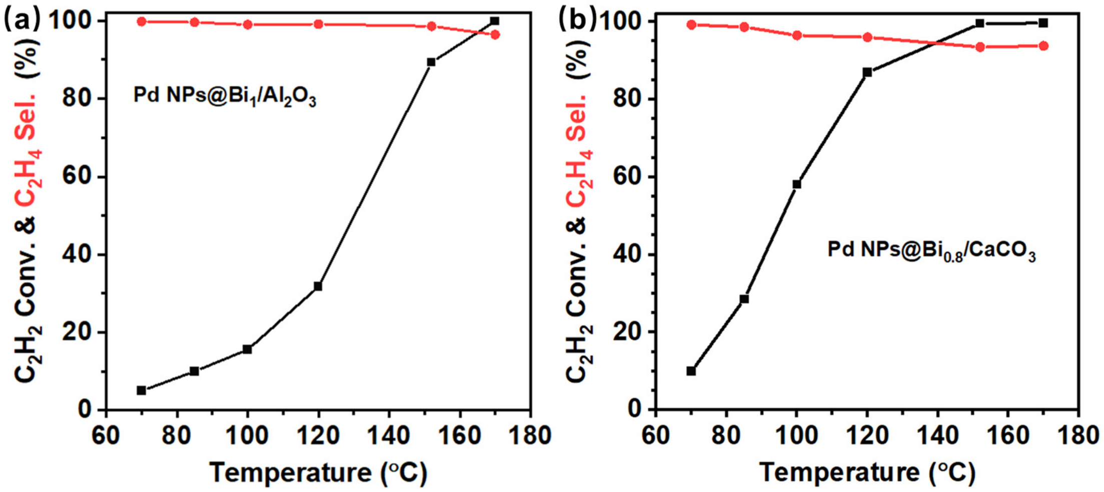

Figure S16 Catalytic performances of Pd NPs@Bi based catalysts in acetylene hydrogenation. (a) C_2H_2 conversion and C_2H_4 selectivity as a function of reaction temperature over Pd NP@Bi_1/Al_2O_3 and (b) C_2H_2 conversion and C_2H_4 selectivity as a function of reaction temperature over Pd NP@$Bi_{0.8}/CaCO_3$. (Reaction conditions: space velocity of 120000 mL h^{-1} g^{-1} and reactant gases: 1.0 vol % C_2H_2, 20.0 vol % C_2H_4, 20.0 vol % H_2, and 59 vol % N_2).
